# Biotherapy of Brain Tumors with Phosphatidylserine-Targeted Radioiodinated SapC-DOPS Nanovesicles

**DOI:** 10.3390/cells9091960

**Published:** 2020-08-25

**Authors:** Harold W. Davis, Subrahmanya D. Vallabhapurapu, Zhengtao Chu, Michael A. Wyder, Kenneth D. Greis, Venette Fannin, Ying Sun, Pankaj B. Desai, Koon Y. Pak, Brian D. Gray, Xiaoyang Qi

**Affiliations:** 1 Division of Hematology/Oncology, Department of Internal Medicine, University of Cincinnati College of Medicine, Cincinnati, OH 45267, USA; harold.davis19@gmail.com (H.W.D.); vallabsa@ucmail.uc.edu (S.D.V.); chu.zhengtao@gmail.com (Z.C.); 2 Department of Cancer Biology, University of Cincinnati College of Medicine, Cincinnati, OH 45267, USA; wyderma@ucmail.uc.edu (M.A.W.); ken.greis@uc.edu (K.D.G.); 3 Division of Human Genetics, Cincinnati Children’s Hospital Medical Center, Cincinnati, OH 45229, USA; venette.davis@cchmc.org (V.F.); ying.sun@cchmc.org (Y.S.); 4 Department of Pediatrics, University of Cincinnati College of Medicine, Cincinnati, OH 45267, USA; 5 The James L. Winkle College of Pharmacy, University of Cincinnati College of Medicine, Cincinnati, OH 45267, USA; pankaj.desai@uc.edu; 6 Molecular Targeting Technologies, Inc., West Chester, PA 19380, USA; cpak@mtarget.com (K.Y.P.); briangray@mtarget.com (B.D.G.); 7 Department of Pathology and Laboratory Medicine, University of Cincinnati, College of Medicine, Cincinnati, OH 45267, USA; 8 Department of Biomedical Engineering, College of Engineering and Applied Science, University of Cincinnati, Cincinnati, OH 45219, USA

**Keywords:** SapC-DOPS nanovesicles, radiation therapy, brain tumor, glioblastoma multiforme, iodination, phosphatidylserine, cancer biomarker, blood–brain barrier, brain-targeted delivery system, brain cancer survival

## Abstract

Glioblastoma multiforme (GBM), a common type of brain cancer, has a very poor prognosis. In general, viable GBM cells exhibit elevated phosphatidylserine (PS) on their membrane surface compared to healthy cells. We have developed a drug, saposin C-dioleoylphosphatidylserine (SapC-DOPS), that selectively targets cancer cells by honing in on this surface PS. To examine whether SapC-DOPS, a stable, blood–brain barrier-penetrable nanovesicle, could be an effective delivery system for precise targeted therapy of radiation, we iodinated several carbocyanine-based fluorescent reporters with either stable iodine (^127^I) or radioactive isotopes (^125^I and ^131^I). While all of the compounds, when incorporated into the SapC-DOPS delivery system, were taken up by human GBM cell lines, we chose the two that best accumulated in the cells (DiI (22,3) and DiD (16,16)). Pharmacokinetics were conducted with ^125^I-labeled compounds and indicated that DiI (22,3)-SapC-DOPS had a time to peak in the blood of 0.66 h and an elimination half-life of 8.4 h. These values were 4 h and 11.5 h, respectively, for DiD (16,16)-SapC-DOPS. Adult nude mice with GBM cells implanted in their brains were treated with ^131^I-DID (16,16)-SapC-DOPS. Mice receiving the radionuclide survived nearly 50% longer than the control groups. These data suggest a potential novel, personalized treatment for a devastating brain disease.

## 1. Introduction

It is estimated that nearly 70,000 new cases of brain tumor are diagnosed in the US each year and they have a dismal prognosis with a five-year survival rate of only 30% [1]. Glioblastoma multiforme (GBM), the most aggressive form of primary brain tumor, is largely drug-resistant [2,3] and treatment is aggravated by protection of the tumor behind the blood–brain barrier (BBB) [4,5]. As such, surgery is the first step for treatment of GBM, which can lead to a reduction of 99% of the tumor. However, GBM cells are generally widely spread throughout the brain at diagnosis, and despite resection of all obvious tumor, most patients with GBM later develop recurrent tumors either near the original site or at more distant locations within the brain [6]. Subsequent to surgery, radiotherapy is routine, and this has been shown to double life expectancy [7,8]. Surgery and radiation are typically combined with chemotherapy, with temozolomide (TMZ) being the drug of choice. While most studies have shown little benefit from the addition of chemotherapy, a large clinical trial randomized to standard radiation versus radiation plus TMZ indicated that the group receiving the combination survived a median of 14.6 months compared to 12.1 months for the group receiving radiation alone [9,10]. Thus, this treatment regimen is now standard of care for most cases of GBM. Despite the use of surgery, radiation and chemotherapy, the median survival for GBM patients is less than 15 months [3,10]. However, a new protocol involving the use of accelerated hypofractionated radiation therapy (60 Gy in 20 fractions extended the median survival to 22 months [11]) and the novel use of alternating electric fields (tumor treating fields; TTF) was able to achieve a 20.9 month median survival with minimal side effects [12]. Nevertheless, new treatment modalities are needed to improve survival.

Delivering chemotherapy with nanovesicles is an emerging concept; however, while these can cross the BBB, intrinsic anti-tumor effects are usually lacking [13,14]. We have developed a nanovesicle, saposin C-dioleoylphosphatidylserine (SapC-DOPS), that is therapeutic against a variety of cancer types with efficacy directly correlated to surface phosphatidylserine (PS) [15,16,17,18,19,20,21,22]. SapC is a lysosomal protein ubiquitous in all cells that has affinity and remarkable specificity for phosphatidylserine (PS) and functions to catabolize glycosphingolipids [23]. When SapC is coupled with DOPS, stable nanovesicles of approximately 200 nm are formed which selectively fuse with cancer cells since, compared to normal cells, viable cancer cells and their associated vasculature commonly have elevated external phosphatidylserine [19,24]. In addition, the tumor microenvironment is acidic [25], which enhances the binding of SapC-DOPS to the surface PS of cancer cells. SapC-DOPS specifically targets PS on the surface of tumor cells in this acidic milieu, leading to ceramide accumulation, caspase activation and eventual apoptosis [15], selectively killing GBM tumor cells, without apparent off-target toxicity to normal cells and tissues [26,27,28]. Indeed, SapC-DOPS has shown an exemplary safety profile in Phase I clinical trials [29,30]. A synergistic effect of SapC-DOPS and TMZ has been demonstrated for GBM in mice [31]. In addition, we have recently demonstrated that SapC-DOPS can cross the BBB to deliver a deficient enzyme and effectively treat a mouse model of Gaucher disease [32]. Importantly, we have recently demonstrated that radiation increases surface PS on a variety of cancer cells and that this enhances the killing of the cells by SapC-DOPS both in vitro and in subcutaneous tumors in a combination therapeutic approach [33]. Crucially, radiation did not increase surface PS on normal cells [33] so a significant impact on SapC-DOPS targeting to these cells would not be expected. As intravenously administered SapC-DOPS nanovesicles are capable of delivering fluorescent probes and magnetic resonance contrast agents directly into the tumor tissue [17,34], we examined whether SapC-DOPS could serve as a carrier for radiation and thus provide both a cytotoxic drug and radiotherapy in one modality. We radiolabeled carbocyanine-based fluorescent probes with ^125^I or ^131^I and incorporated these into SapC-DOPS nanovesicles. We then injected these radioiodine-labeled nanovesicles into mice and recorded the pharmacokinetics (PK) and the efficacy for treatment of GBM. ^131^I was selected for our efficacy studies since it is an FDA-approved radionuclide currently used as a therapy for thyroid cancer [35], its half-life is short (eight days), its β and γ emissions are useful for both radiotherapy and imaging applications over several days, and treatment with β-particle-emitting radionuclides is the preferred approach for the management of bulky and heterogeneous tumors [36,37]. Since SapC-DOPS crosses the BBB and targets surface PS on tumors, the ^131^I-labeled nanovesicles may be a useful tool in the arsenal for personalized and highly localized brain cancer therapy.

## 2. Materials and Methods

### 2.1. SapC Preparation and Purification

SapC is produced using the pET expression system in *Escherichia coli* cells and HPLC purified by the Changji Bio-Tech Company (Changzhou, China) as previously described with modification [38]. SapC preparation is conducted under Good Manufacturing Practices (GMP).

### 2.2. Synthesis and Characterization of Iodinated SapC-DOPS Nanovesicles

**Figure 1:**
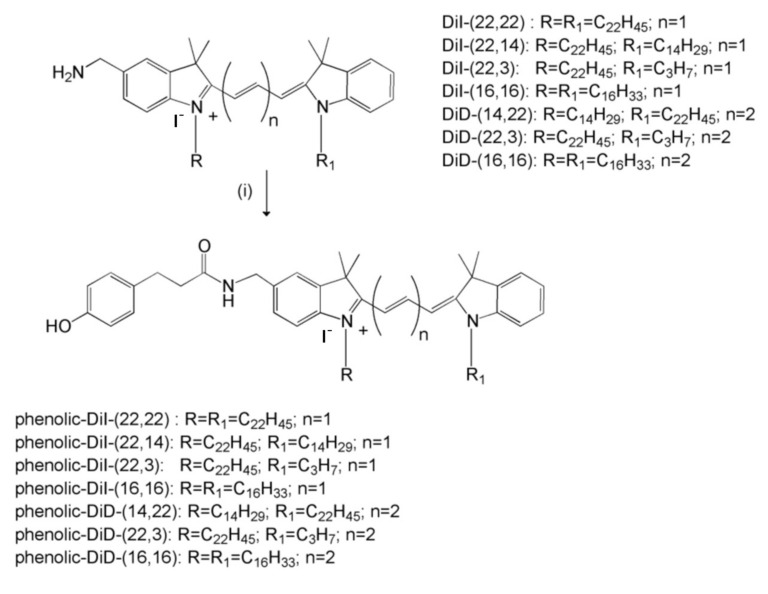
Structures of carbocyanine-based fluorescent probes and their phenolic derivatives. The carbocyanine-based compounds were prepared as in Materials and Methods. These were then treated with the Bolton-Hunter reagent to provide the corresponding phenolic derivatives.

The amino-functionalized carbocyanine-based fluorescent reporters DiD-(14,22), DiD-(22,3), DiD-(16,16), DiI-(22,22), DiI-(22,14), DiI-(22,3) and DiI-(16,16) with diverse hydrocarbon tail configurations were prepared using previously described procedures [39]. The amino-functionalized dyes were then treated with the Bolton-Hunter reagent, n-succinimidyl-3-(4-hydroxyphenyl) propionate, to provide the corresponding phenolic substituent analogs (Figure 1). Phenolic derivatives were characterized by a combination of proton NMR, low resolution MS, HPLC, UV-VIS and fluorescence properties (Table 1). All spectroscopic data obtained was consistent with data for known compounds belonging to the same family of cyanine dyes.

The diverse hydrocarbon tail configurations were tested for optimal nanovesicle incorporation and retention. Six compounds were iodinated with iodine-127 (non-radioactive, stable isotope) and inserted into SapC-DOPS as previously described [15].

### 2.3. Iodination of Phenolic Compounds with Iodine-127

We used two methods to iodinate the compounds. Method 1 was carried out using Iodogen precoated iodination tubes (Pierce, Rockford, IL, USA) according to the manufacturer’s suggested (direct) procedure with some modifications. For cold labeling procedures, 100 μg of compounds (only DiI compounds were used with this method) in tertiary butyl alcohol (TBA; 10 mg/mL stock solutions) was added to TBA in a glass test tube, and NaI in phosphate-buffered saline (PBS) was then added in a total reaction volume of 100 μL. TBA 100% or a mix of TBA:PBS cosolvent ratios involving 95% TBA, 85% TBA, 75% TBA, 50%TBA or 25% TBA were tested. Final NaI was 1 mM. The mixture was then briefly vortexed, added to the iodination tube (Iodogen tubes, Pierce, Dallas, TX, USA), pre-rinsed with PBS, and the reaction mix was incubated for 20 min at room temperature with periodic shaking. Labeled compounds were recovered by elution through Sep-Pak (C4 reverse phase, Perkin Elmer Cat# N9306590, Waltham, MA, USA) columns by sequential addition of 1 mL methanol, 1 mL PBS, iodinated sample, 1 mL PBS and 0.4 mL chloroform:methanol (1:2). The eluate was vacuum-dried and resuspended in 100 μL ethanol. Subsequent iodination reactions with a new batch of Iodogen-coated tubes led to inconsistent iodination results. It was eventually determined that the presence of an excess of Iodogen in the reaction resulted in decomposition of the phenolic dyes. A solution phase procedure where the amount of Iodogen present could be controlled was therefore studied to overcome this technical hurdle.

**Figure 2:**
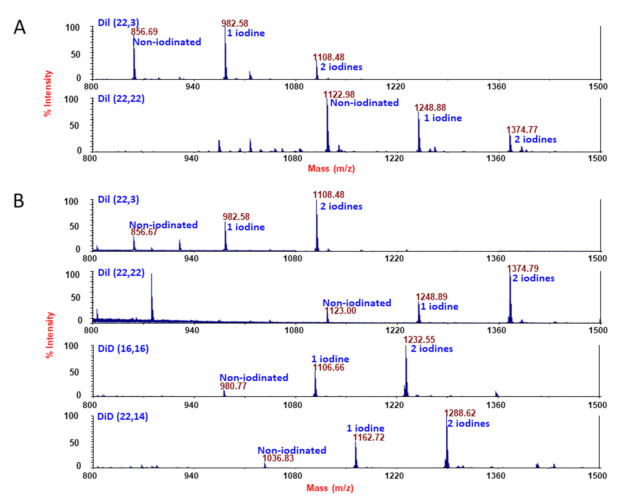
Iodination of phenolic DiI and DiD compounds. (**A**) Iodination with NaI was carried out using Iodogen precoated iodination as the first method described in Materials and Methods. The optimal ratios of tertiary butyl alcohol to phosphate-buffered saline (PBS) were 50:50 for DiI (22,3) and 75:25 for DiI (22,22). Shown are the mass spectra acquired as positive ion reflector mode on a Sciex 4800 matrix-assisted laser desorption/ionization (MALDI) time-of-flight (TOF)/TOF instrument. (**B**) Iodination with NaI was carried out using Iodogen in solution as the second method described in Materials and Methods. Shown are the mass spectra acquired as in Figure 1.

Therefore, for Method 2, phenolic compounds (100 μg) were dissolved in *N*,*N*-dimethylformamide (DMF; Sigma, St. Louis, MO, USA) to obtain a concentration of 1 µg/µL. Another 100 µL DMF was added, then 1 mM NaI was transferred to the tube and the original vial was rinsed several times with water to a final volume of 400 μL, all added to the test tube. Finally, 50 µL of Iodogen (116 nmoles 1,3,4,6-tetrachloro-3α-6α-diphenylglycouril; Sigma, St. Louis, MO, USA) from a 1 mg/mL stock in DMF was added. After 2 min vortexing, the reaction was quenched by addition of 30 µL of sodium bisulfite solution from a 10 mM stock solution (300 nmoles, made in purged HPLC water. After addition of 1 mL water and vortexing, the contents were transferred to a new glass tube; the first tube was rinsed twice with 1 mL chloroform and this was added to the reaction mixture. Phase separation was obtained by vortexing. The upper aqueous phase was removed and the lower phase was dried under nitrogen gas; the dried film was dissolved in 200 µL ethanol. To verify the degree of mass shift consistent with iodination status, samples were diluted 1:5, 1:10 and 1:20 in matrix-assisted laser desorption/ionization (MALDI) matrix (5 mg/mL α-cyano-4-hydroxycinnamic acid in 10 mM ammonium phosphate, 60% acetonitrile/0.1% formic acid) and spotted for MALDI-TOF (time of flight) analysis. All spectra were acquired in positive ion reflector mode on a Sciex 4800 MALDI TOF/TOF instrument. For comparison of the two methods, see the mass spectrometric runs in Figure 2.

### 2.4. Incorporation of the Compounds into SapC-DOPS and Targeting GBM Cells

**Figure 3:**
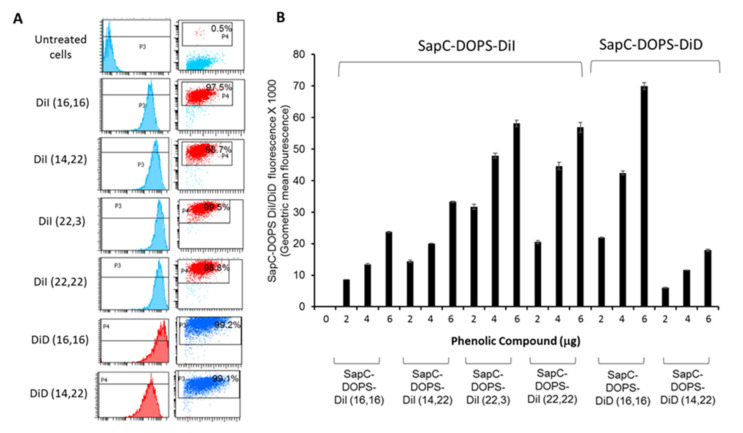
Saposin C-dioleoylphosphatidylserine (SapC-DOPS) nanovesicles assembled with the iodinated phenolic DiI/DiD compounds robustly target U87Δ epidermal growth factor receptor (EGFR) GBM cells. (**A**) The left row of panels shows the histograms and the right row of panels shows dot plots with the compounds internalized by the cells. U87ΔEGFR cells treated with 6 μg of the indicated SapC-DOPS DiI/DiD compounds for 3 h exhibit robust fluorescence as evident by flow cytometry. Untreated cells were used as controls. (**B**) Graphical representation of flow cytometry data indicating the fluorescence of the cells loaded with 2, 4 or 6 μg of the dyes.

**Figure 4:**
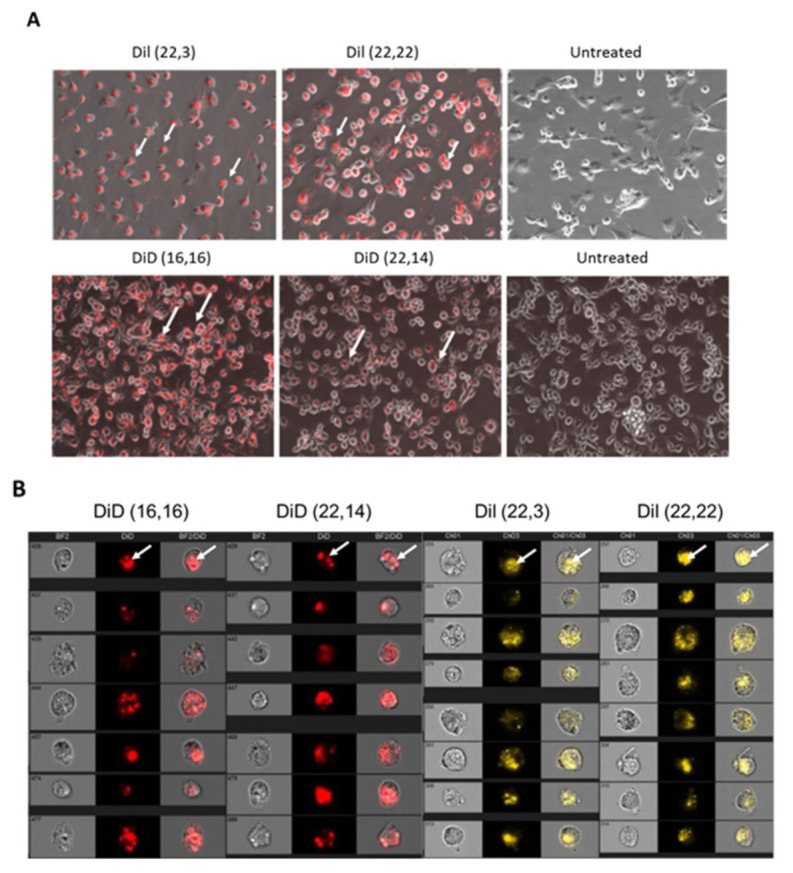
SapC-DOPS nanovesicles assembled with the iodinated phenolic DiI or DiD compounds robustly target GBM cells and are internalized. (**A**) U87ΔEGFR cells were incubated with 6 μg of the compounds for 3 h then examined with fluorescent microscopy. Note the red fluorescence (arrows) of iodinated phenolic compound SapC-DOPS in the left and middle panels compared to the untreated cells in the right panels. (**B**) U87ΔEGFR cells treated for 3 h with 6 μg of the indicated compounds were washed and analyzed by ImageStream flow cytometry for single cell visualization. Arrows show the internalized DiI/DiD fluorescence signal. The first column for each compound shows the bright field image of the cells; the second column is the fluorescence (red for DiD and yellow for DiI) of the compound and the third is the overlay showing that the compound is internalized.

The six phenolic compounds were incorporated into SapC-DOPS nanovesicles as previously described [15,27]. The fluorescent nanovesicles were then tested for association with human U87Δ epidermal growth factor receptor (EGFR)-Luc GBM cells; these cells express a truncated, constitutively active, mutant epidermal growth factor receptor (EGFRvIII, obtained from Dr. Webster Cavenee; Ludwig Cancer Institute, San Diego, CA, USA) by flow cytometry using a BD Fortessa (Figure 3). The reporter compounds that associated most robustly with the GBM cells were phenolic DiI-(22,22), phenolic DiI-(22,3), phenolic DiD-(14,22) and phenolic DiD-(16,16). These compounds were further tested by fluorescence microscopy (Keyence BIOREVO BZ-9000, Itasca, IL, USA) and by Amnis^®^, ImageStreamX Mark II (Luminex, Northbrook, IL, USA) imaging flow cytometry (Figure 4) to determine if they were internalized into the cells. These data indicated that all of the compounds, when incorporated into SapC-DOPS nanovesicles, were taken up by cells. We chose the DiI and DiD compounds that were best internalized to conduct pharmacokinetic (PK) and biodistribution studies in mice: DiI (22,3) and DiD (16,16).

### 2.5. Radioiodination of the Compounds

Phenolic DiI (22,3) or DiD (16,16) was dissolved in *N*,*N*-dimethylformamide (DMF; Sigma) to obtain a concentration of 1 µg/µL. Radioiodination was carried out by addition of 100 µL of phenolic compound from 1 mg/mL stock solution in DMF to a glass tube. Another 100 µL DMF was added. Then the ^125/131^INa was transferred to the tube and the original vial was rinsed several times with water to a final volume of 400 μL, all added to the test tube (25 mCi of ^125^INa (PK studies) or 37.6 mCi (efficacy studies) of ^131^INa). Iodine isotopes in ~50 μL of 100 mM NaOH were obtained from Perkin Elmer. Iodination was achieved by the Method 2 described above.

### 2.6. Analysis of ^131^I Incorporation into Phenolic DiD (16,16) by Thin Layer Chromatography (TLC)

Based on the results of the PK studies, we conducted the efficacy studies with DiD (16,16) and further tested this compound for labeling with the therapeutic isotope, ^131^I. When 37.6 mCi of ^131^I Na was used in the iodination reaction with phenolic DiD (16,16), the chloroform phase contained a measured radioactivity of 5.91 mCi (15.9% of activity used), with a loss of 17.92 mCi in the upper phase and 14.03 mCi in the interphase, during the phase separation step. However, when 14.0 mCi of ^131^I Na was used, the chloroform phase contained a measured radioactivity of 10.74 mCi (77.1% of activity used), with a loss of 1.69 mCi in the upper phase and 0.75 mCi in the interphase. In each case, all of the blue color from phenolic DiD (16,16) and iodinated phenolic DiD (16,16) was in the organic phase.

**Figure 5:**
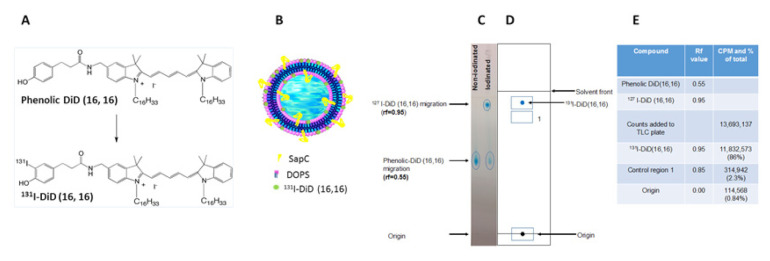
Thin layer chromatography (TLC) schematic depiction of ^127^I-DiD (16,16) and ^131^I-DiD (16,16) samples. (**A**) Schematic of iodination of DiD (16,16). (**B**) ^131^I-DiD (16,16)-SapC-DOPS model. (**C**) Non-iodinated and ^127^I-DiD (16,16) samples; 1 µg of each were spotted at the origin of the TLC plate. Ovals indicate the migration of indicated samples. (**D**) Schematic representation of ^131^I-DiD (16,16) migration. Squares represent scraped regions from TLC plate. (**E**) Table showing the retention factor (Rf) values of non-iodinated and ^127^I-DiD (16,16) samples and counts per minute (CPM) for ^131^I-DiD (16,16).

For each of DiD (16,16), ^127^I-iodinated DiD (16,16) or ^131^I-iodinated DiD (16,16), 1 µg was loaded onto TLC silica gel 60 plates (Millipore, Burlington, MA, USA) and air dried. Chloroform (28.1%), ethanol (32.7%), triethylamine (32.7%) and water (6.5%) were used as the solvent for migration of the materials. The retention factors (Rf) of the DiD (16,16) and the ^127^I-DiD (16,16) were calculated as a ratio of the migration of the compound from the origin and the distance the solvent front moved (Figure 5).

### 2.7. Assembly of SapC-DOPS Nanovesicles with ^131^I Radioiodinated Phenolic DiD (16,16)

Two batches of SapC-DOPS nanovesicles with ^131^I radioiodinated phenolic DiD (16,16) were prepared by adding 0.89 mCi of ^131^I-radioiodinated DiD (16,16) in ethanol and 730 µg of DOPS in chloroform (10 mg/mL) in a glass test tube, and organic solvents were dried under nitrogen gas. Next, 1.2 mg of saposin C protein in 20 µL of citrate phosphate buffer (pH 5.0) was added to the dried compounds. After addition of PBS (1 mL), the dried film and the mixture were bath sonicated in a Branson sonicator containing ice water for 30 min. SapC-DOPS nanovesicles assembled with ^131^I-iodinated phenolic DiD (16,16) were separated from non-assembled compounds by passing the reaction mixture through PD-10 columns (GE Healthcare, Piscataway, NJ, USA). Vesicles were eluted with PBS. Eluates containing the radioiodinated compounds assembled with SapC-DOPS were collected. At each step, radioactivity was measured with a gamma counter. Eluted vesicles of ^131^I-phenolic DiD (16,16)-SapC-DOPS contained 0.367 mCi (41.2% recovery) and 0.405 mCi (45.5% recovery) from each column, with the remaining portion adhered to the columns.

### 2.8. Pharmacokinetic Studies with ^125^I-DiI (22,3)- and ^125^I-DiD (16,16)-SapC-DOPS

All animal studies were approved by the University of Cincinnati Institutional Animal Care and Use Committee (IACUC number 11-05-05-02). Three nude mice (Taconic Biosciences, Rensselaer, NY, USA) were used for each compound in SapC-DOPS and were injected intravenously into the tail vein. Blood was collected from the saphenous vein at 0, 0.5, 2, 4, 6 and 24 h following injection and 20 μL was counted for radioactivity. After the 24 h blood collection, the mice were euthanized by CO_2_ rebreathing and cervical dislocation.

### 2.9. Efficacy Studies with ^131^I-DiD (16,16)-SapC-DOPS

Twenty-four nude mice (12 males and 12 females from Taconic Biosciences) were injected with 1 × 10^5^ U87ΔEGFR-Luc into the right frontal cortex just laterally to the sagittal suture [17]. On day 2, the mice (6/group) were injected with PBS, ^127^I-DiD (16,16)-SapC-DOPS or ^131^I-DiD (16,16)-SapC-DOPS. The typical therapeutic dose was 4 mg/kg of SapC and 2 mg/kg of DOPS, a dose we have previously used for evaluation of chemotherapeutic agents on enhancement of the SapC-DOPS effect [18]. Dosing was repeated on days 4, 6 and 9. The amount of DiD (16,16) was adjusted daily to account for radioactive decay (for both the radiolabeled and unlabeled compound). The drug (−200 μL) was administered intravenously into the tail vein. The mice received 60–200 μCi of the ^131^I-DiD (16,16)-SapC-DOPS per injection. The radiation dose was determined by measuring gamma radiation of the syringe before and after the injection. A SapC-DOPS dose response was conducted with 8 mice per group. We chose a dose of SapC-DOPS with minimal benefit for the radiation studies so that we could determine if targeted dosing of ^131^I could enhance survival. Mice were followed until death or until they lost 20% of their body weight, at which time they were euthanized by CO_2_ rebreathing and cervical dislocation.

## 3. Results

### 3.1. Chemical and Physical Properties of the Iodinated Compounds

Iodination of the compounds was much more efficient with Method 2, when Iodogen was in solution. Method 2 provided more consistent results than Method 1 and the yields were better. When the compounds were iodinated by this method, the non-iodinated peak was much smaller and the compounds were more likely to incorporate two iodines per molecule (compare Figure 2A,B).

### 3.2. Phenolic Dye Uptake by Brain Tumor Cells and Pharmacokinetics

We then tested the ability of these compounds to interact with GBM U87ΔEGFR cells. While all of the compounds were internalized by the U87ΔEGFR cells (Figure 3 and Figure 4), we chose to further evaluate DiI (22,3) and DiD (16,16) as their uptakes were particularly efficient. For pharmacokinetic studies, normal (no tumor) nude mice were injected with the ^125^I-labeled compounds, and blood was drawn and counted for radioactivity at 0, 0.5, 2, 4, 6 and 24 h. We used ^125^I for the PK studies since it has a much longer half-life than ^131^I (60 days vs eight days, respectively) so we did not have to adjust for decay during the tests. As shown in Table 2, the PKs were similar for the two compounds but DiD (16,16) had a slower time to peak (4 h vs 1 h) and a longer elimination half-life (11.5 h vs 8.4 h) compared to DiI (22,3). The PK data convinced us to proceed with DiD (16,16) for efficacy studies.

For analyses of the radioiodination efficiency of ^131^I-iodinated DiD (16,16), a band corresponding to the blue dye (DiD 16,16) with Rf value 0.95 and other bands corresponding to Rf values of 0.85 and 0 (the origin) were scraped from the TLC plate and radioactivity was measured with a gamma counter (Figure 5).

Radioanalyses of the phase separated ^131^I-DiD (16,16) from the 14 mCi reaction by TLC revealed that 86% of the ^131^I was in the phenolic DiD (16,16) band (Rf = 0.95). Therefore, the percentage of radioactivity that ended up in the product (i.e., radiolabeling yield) was 58.8%. Furthermore, the presence of only a small percentage of radioactivity in the TLC bands corresponding to the origin and to the Rf value of 0.85 (0.84% and 2.3%, respectively) suggest minimal presence of free ^131^I in the solution containing radioiodinated DiD (16,16) (Figure 5).

### 3.3. Radioiodinated SapC-DOPS Improved Survival of Brain Tumor-Bearing Mice

**Figure 6:**
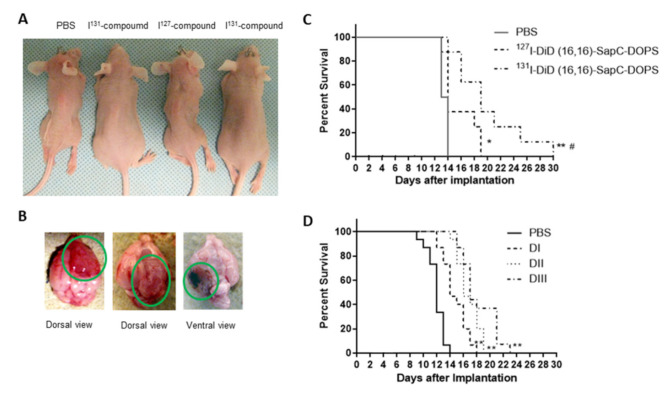
Physical characteristics of glioblastoma multiforme (GBM)-bearing mice after treatments. (**A**) Whole body images of live anesthetized mice 13 days after implantation of the GBM tumor cells (1 × 10^5^ U87ΔEGFR-Luc). All of the mice had tumors. The control mice died within a day of when these pictures were taken. (**B**) Isolated brains showing GBM tumors following euthanasia of moribund mice. Tumors were similar in each group. (**C**) Mouse survival from glioblastomas after treatment. Two days after implantation of tumor cells, the mice (6/group) began treatment with PBS, ^127^I-DiD (16,16)-SapC-DOPS or ^131^I-DiD (16,16)-SapC-DOPS (SapC = 4 mg/kg, DOPS = 2 mg/kg). The treatments with the ^131^I doses were 61, 68, 177 and 93 μCi. Mice were euthanized when they became moribund. * *p* = 0.0327, PBS vs ^127^I-DiD (16,16)-SapC-DOPS; ** *p* = 0.0004, PBS vs. ^131^I-DiD (16,16)-SapC-DOPS; ^#^ *p* = 0.0378, ^127^I-DiD (16,16)-SapC-DOPS vs ^131^I-DiD (16,16)-SapC-DOPS by log-rank test. (**D**) Dose response of GBM tumors to SapC-DOPS. Mice (8/group) were injected with U87ΔEGFR-Luc (1 × 10^5^ cells/mouse) as in A–C with SapC-DOPS treatments started on day 4 and the mice were injected with SapC-DOPS 2 times a week. DI: SapC = 2.7 mg/kg, DOPS = 1.3 mg/kg; DII: SapC = 8 mg/kg, DOPS = 4 mg/kg; DIII: SapC = 24 mg/kg, DOPS = 12 mg/kg. ** *p* < 0.001 by log-rank test for each dose (*n* = 6 for each group).

GBM cells were implanted into nude mouse brains and treatment was started two days later. Mice were injected with PBS, ^127^I-DiD (16,16)-SapC-DOPS or ^131^I-DiD (16,16)-SapC-DOPS. The SapC-DOPS dose was approximately 4 mg/kg of SapC and 2 mg/kg of DOPS for both ^127^I- and ^131^I-receiving animals. While the mice lost weight as the tumors grew (Figure 6A), there was also weight loss due to the radiation, especially after the higher dose given on day 6. Glioblastoma often causes cachexia and it is not surprising that the high dose of radiation given on day 6 induced acute radiation sickness. Importantly, the mice that received ^131^I-DiD (16,16)-SapC-DOPS did not lose weight as quickly (except after day 6 when they received the 177 μCi dose of ^131^I) as the other mice. These mice also survived (median survival ID_50_ = 20 days) longer than those that received ^127^I-DiD (16,16)-SapC-DOPS (ID_50_ = 14 days; improvement of > 43%; *p* = 0.0378) or PBS (ID_50_ = 13 days; improvement of > 48%; *p* = 0.0004) (Figure 6C). The last mouse that received ^131^I-DiD (16,16)-SapC-DOPS died on day 30 after implantation. All of the mice had GBMs (Figure 6B) at the time of death.

For these studies, we used a dose of SapC-DOPS that has minimal effect (Figure 6D) to determine if the addition of ^131^I would significantly improve lifespan for “proof of concept”. Preliminary studies indicated that labeling of SapC-DOPS with CellVue Maroon (CVM), a close structural analog of DID (16,16), did not alter the targeting of SapC-DOPS to the GBM tumor [27,28], and non-radioactive iodine (^127^I) had no effect on the efficacy of SapC-DOPS in treating GBM (compare Figure 6C,D) and thus a non-iodine labeled group was not included in this experiment. Interestingly, when we examined another group of mice that did not have brain tumors, the accumulation of ^131^I in those brains had a 3.7-fold lower %ID/g at 24 h than brains from our efficacy study with GBMs. This was similar to what we detected earlier with ^125^I-labeled SapC-DOPS [27].

## 4. Discussion

By targeting PS-rich domains on the cancer cell membrane, SapC-DOPS selectively kills GBM tumor cells both in vivo and in vitro, without apparent off-target toxicity to normal cells and tissues [15,19,26,27,28,40,41]. The cytotoxicity of SapC-DOPS is positively correlated with cancer cell surface PS but also requires an acidic environment; thus it does not kill normal tissues. We have previously demonstrated that contrast reagents can also be loaded into SapC-DOPS for magnetic resonance imaging (MRI) or positron emission tomography (PET) on mice [18]. Since radiation is a critical component of GBM treatment, we investigated whether loading SapC-DOPS with a radioactive isotope could enhance the effects of the nanovesicle by virtue of the ability of SapC-DOPS to target tumor PS and thus facilitate confinement of radiation to the tumor issue. Free iodine is targeted to the thyroid, the only organ that uses this element, but its radioactive isotopes kill all cells so it is important to limit exposure of non-tumor cells. Encapsulation of ^131^I into SapC-DOPS not only prevents thyroid uptake [27] (and data not shown) but quickly (within 24 h) directs the radioisotope to the GBM tumor. In this study, we have continued our work on the use of iodinated phenolic compounds coupled to SapC-DOPS [27]. Herein, we establish that a revised iodination method allows for additional binding of iodines, and therefore, more radiation to be delivered to the tumor. We have recently shown that repeated irradiation of cells leads to higher surface PS and that this enhanced the effects of SapC-DOPS on a number of cancer cell types [33]. Although we did not measure surface PS of the brain tumors in this study, treatment of mice bearing GBMs with ^131^I-DiD (16,16)-SapC-DOPS significantly improved survival compared with ^127^I-DiD (16,16)-SapC-DOPS. While there is an initial weight loss, probably due to the radiation sickness all of the mice, regardless of treatment regimen, eventually lost about the same amount of weight, albeit at a later date for the ^131^I-treated mice (data not shown). We have previously demonstrated that SapC-DOPS has limited toxicity both in mice [17,18] and humans [29,30]. In addition, we have used a structural analog of DiD, the fluorescent compound, CVM, to label SapC-DOPS in many studies and have not detected any change in behavior of mice treated with SapC-DOPS-CVM compared to unlabeled SapC-DOPS [18,27].

Although the efficacy of the combination therapy (SapC-DOPS and ^131^I) is highly statistically significant, it is not clinically exceptional due to lack of dose optimization. To enhance these results, we will need to increase the doses of SapC-DOPS and determine an optimal dose of ^131^I-DiD (16,16). We have previously demonstrated that high frequency of administration of SapC-DOPS prolongs survival [18] so more doses may be required. As shown in Figure 6D, there is a dose dependence with SapC-DOPS. We have not used higher doses than 8–12 mg/kg SapC and 4–12 mg/kg DOPS in mice but this can probably be increased since there have been no dose-limiting toxicities in human studies [29,30] and in some studies we have used a much more frequent dosing schedule [18]. More importantly, the dose of ^131^I-DiD (16,16) needs to be adjusted to minimize toxic effects while maximizing tumor killing. This can be done by changing the amount of ^131^I used to iodinate the DiD (16,16) or by altering the ^131^I-DiD (16,16) incorporated into the SapC-DOPS nanovesicles. Tumors are heterogeneous for many characteristics, including surface PS [41], and while most cancer cells have elevated cell surface PS, this varies even within a distinct cell line [19,24]. Our previous data suggest that radiation targets low surface PS cells while SapC-DOPS works better on higher surface PS cells, so a combination of these modalities should result in enhanced tumor cell death [33]. Moreover, radiation-induced surface PS increase may further amplify the efficacy of SapC-DOPS tumor cytotoxicity. We expect that we can titrate the ^131^I to maximize the efficacy while minimizing the radiation-induced toxicity. In addition, once the initial effects of the radiation are accrued (i.e., killing low surface PS cells), the dosing regimen can be continued without the ^131^I as we and others have revealed that repeated radiation treatment leads to radiation resistance [33,42].

## 5. Conclusions

**Figure 7:**
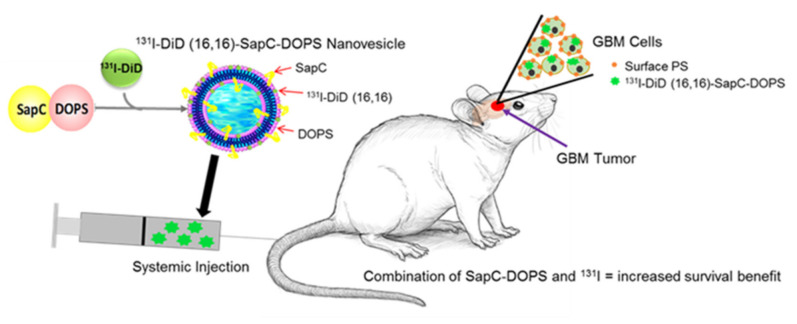
^131^I-DiD (16,16)-SapC-DOPS increases GBM-bearing mouse survival. SapC-DOPS was prepared with the radioiodinated phenolic compound DiD (16,16). The resulting modality was injected into mice bearing a GBM. We propose that the radiation from the ^131^I kills tumor cells with low surface phosphatidylserine (PS) while the SapC-DOPS kills predominately tumor cells with higher surface PS; thus there is an enhancement in treatment of the GBM.

Our novel use of nanovesicle transport of radiation to a tumor indicates that SapC-DOPS can deliver ^131^I to mouse glioblastomas. Since the elimination half-life for ^131^I-DiD (16,16)-SapC-DOPS is short (11.5 h), normal tissues should have minimal exposure. SapC-DOPS targets cancer cell surface PS so it can be personalized to individual tumors following biomarker (tumor surface PS) assessment. We conducted these pilot studies to establish the practicality of this treatment regimen but future studies will be required to determine the precise dosage of the ^131^I-DiD (16,16)-SapC-DOPS. We believe that incorporation of ^131^I into the nanovesicles will allow for targeting of radiation to the tumor with greatly reduced exposure of the surrounding normal tissue, to improve safety and convenience of treating intracranial neoplasms (Figure 7).

## Figures and Tables

**Table 1:** Absorbance and fluorescence properties of each phenolic compound. The derivatives were characterized by a combination of proton NMR, low resolution MS, HPLC, UV-VIS and fluorescence properties.

**Synonym**	**^Calc^Mass**	**^Obs^Mass**	**Purity at 260 nm (AUC)**	**Purity at 550 nm (AUC)**	**Retention Time (min)**	**Abs Max (nm)**	**FL Ex Max (nm)**	**ε (M^−1^cm^−1^)**
Phenolic-DiI(22,22)	1123	1123.4	94.60%	100%	16.1	555	571	134,100
Phenolic-DiI(22,14)	1010.8	1010.6	98.50%	100%	14.7	555	571	143,400
Phenolic-DiI(22,3)	856.7	856.3	82.40%	100%	13.6	555	570	118,800
Phenolic-DiI(16,16)	954.8	954.9	90.90%	100%	14.3	556	571	133,100
**Synonym**	**^Calc^Mass**	**^Obs^Mass**	**Purity at 260 nm (AUC)**	**Purity at 650 nm (AUC)**	**Retention Time (min)**	**Abs Max (nm)**	**FL Ex Max (nm)**	**ε (M^−1^cm^−1^)**
Phenolic-DiD(14,22)	1036.9	1036.9	98.10%	99.50%	14.2	652	670	222,200
Phenolic-DiD(22,3)	882.7	882.7	97.40%	100%	13.6	651	671	235,600
Phenolic-DiD(16,16)	980.8	981.6	96.20%	100%	12.8	652	671	227,500

**Table 2:** Pharmacokinetic properties of ^125^I-DiI (22,3)- and DiD (16,16)-SapC-DOPS. The compounds were radioiodinated as described in Materials and Methods, incorporated into SapC-DOPS nanovesicles and injected into nude mice. Blood was taken at 0, 0.5, 2, 4, 6 and 24 h and measured for radioactivity. The numbers in the top row indicate the number of the mouse.

DiI (22,3)						DiD (16,16)					
Pharmacokinetic Parameters	1	2	3	Mean of PK Values	PK Values of Mean Conc	Pharmacokinetic Parameters	1	2	3	Mean of PK Values	PK Values of Mean Conc
Time to Peak (Tmax) (h)	0.5	1	0.5	0.66 ± 0.28	1	Time to Peak (Tmax) (h)	4	4	4	-	4
C_p_max (Peak conc) (cpm/mL)	48.3	35.8	46.1	43.4 ± 6.67	40.1	C_p_max (Peak conc) (cpm/mL)	30.1	26.8	33.7	30.2 ± 3.4	30.23
Elimination HalfLife (h)	10.69	9.58	6.11	8.79 ± 2.38	8.39	Elimination HalfLife (h)	7.27	22.67	8.96	12.96 ± 8.4	11.5
AUC_0-24_ (cpm.hr/mL)	442	204	462	369 ± 143	370	AUC_0-24_ (cpm.h/mL)	446	496	506	482 ± 32	482
AUC_0-t_ (cpm.hr/mL)	552	240	491	427 ± 165	422	AUC_0-t_ (cpm.h/mL)	494	967	600	687 ± 248	624
Volume of Distribution/F	69,424	142,902	44,613	85,646 ± 51,113	71,284	Volume of Distribution/F	52,845	84,271	53,614	63,576 ± 17,925	64,239
Systemic Clearance/F (mL/h)	4500	10,337	5058	6631 ± 3221	5885	Systemic Clearance/F (mL/h)	5033	2575	4147	3918 ± 1244	3991
